# Novel Low-Cost Tracheo-Innominate Artery Fistula Bleed Simulator

**DOI:** 10.7759/cureus.19873

**Published:** 2021-11-24

**Authors:** Renato Rapada, Ryan C Gifford-Hollingsworth, Timoteo R Cadena, Tiffany Nelms, Zachary Sletten

**Affiliations:** 1 Emergency Medicine, San Antonio Military Medical Center, San Antonio, USA; 2 Simulation, San Antonio Military Medical Center, San Antonio, USA; 3 Simulation, Brooke Army Medical Center, San Antonio, USA

**Keywords:** tracheo-innominate fistula, simulation trainer, skills and simulation training, teaching by simulation, critical care simulation, emergency medicine resuscitation, simulation, simulation lab teaching and training, emergency medicine, emergency medicine training

## Abstract

Tracheo-innominate artery fistulas are a rare complication of indwelling tracheotomies with a very high mortality rate. Due to the rare occurrence of this surgical emergency, most medical providers have little to no training or experience in recognizing, stabilizing, and repairing this life-threatening condition. Simulation of rare emergencies helps close knowledge gaps of medical providers at all levels. Although many providers may never experience these emergencies throughout their careers in clinical medicine, it is imperative that they distinguish and apply techniques for temporizing these life-threatening conditions in order to decrease patient mortality. This novel, low-cost, and easy-to-implement simulation is geared towards this goal and has been successfully tested in small group simulations at one academic center in San Antonio, Texas.

## Introduction

Tracheostomies are amongst the oldest reported surgical procedures [1]. Early in the history of this procedure very few attempts were successful and most patients died during or shortly after [1]. Since the 20th-century tracheostomies are utilized with increasing frequency and as of 2014 more than 100,000 tracheostomies are reported annually in the United States alone [1,2]. Indications for tracheostomies include the need for prolonged ventilatory support, need for chronic pulmonary clearance, protection from aspiration, and for bypassing supraglottic and glottic obstructions or stenosis. As this procedure is increasingly utilized, the number of patients presenting with associated complications has increased as well [2]. Although there are several complications associated with tracheostomies ranging from minor to severe, few are as life-threatening as a tracheo-innominate artery fistula (TIF) with a perioperative mortality of greater than 50% [2,3].

A TIF is caused by an erosion of the anterior tracheal wall into the posterior wall of the innominate artery. This erosion is induced by different mechanisms, most commonly by anterior tracheal pressure from an overinflated tracheostomy cuff, but also include local infection, penetrating trauma, cervical orthopedic hardware migration, tracheostomy placement below the level of the third tracheal ring [3]. Once a TIF is formed, it is estimated that 50% of patients will present with a self-limiting sentinel bleed prior to acute hemorrhage [4,5]. If a provider maintains a high index of suspicion for a TIF, it can be identified sooner with direct visualization under bronchoscopy or with CT angiography. Rapid identification by a trained provider can improve patient outcomes as described in recent pediatric literature [6]. Although treatment of a TIF ultimately requires surgical intervention, multiple maneuvers to achieve hemostasis in an acutely hemorrhaging patient have been described. These maneuvers include over-inflation of the tracheostomy tube, endotracheal intubation, and direct digital pressure applied through the trachea anteriorly compressing the innominate artery. These techniques in combination with emergent surgical intervention, massive transfusion, and reversal of coagulopathy increase the patient’s chances of survival if instituted early.

TIFs are an exceedingly rare complication (0.1-1%) associated with high mortality, which may be significantly decreased with early identification and rapid intervention [7-9]. As most providers will not encounter this life-threatening surgical emergency during their formal training years, this makes a simulator an ideal candidate for simulation-based training to fill in the knowledge gaps and provide hands-on training in the above-described maneuvers [10-12]. The authors present a novel, low-cost, and easy-to-implement TIF bleed simulation geared towards this goal.

## Technical report

This simulation was built with a SimMan® 3G manikin (Laerdal Medical, Stavanger, Norway); however, other manikin models may be used in its place. The following steps and figures serve to outline the simple process of configuring the TIF bleed simulator. 

**Figure 1:**
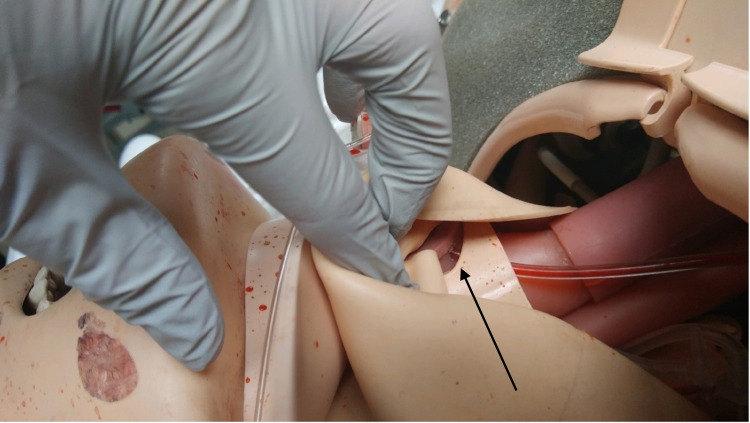
Intravenous tubing passing through drilled hole just below flange into simulated trachea (arrow)

A small hole should be drilled through the simulated trachea just wide enough to pass the IV tubing as can be seen in Figure 1.

**Figure 2:**
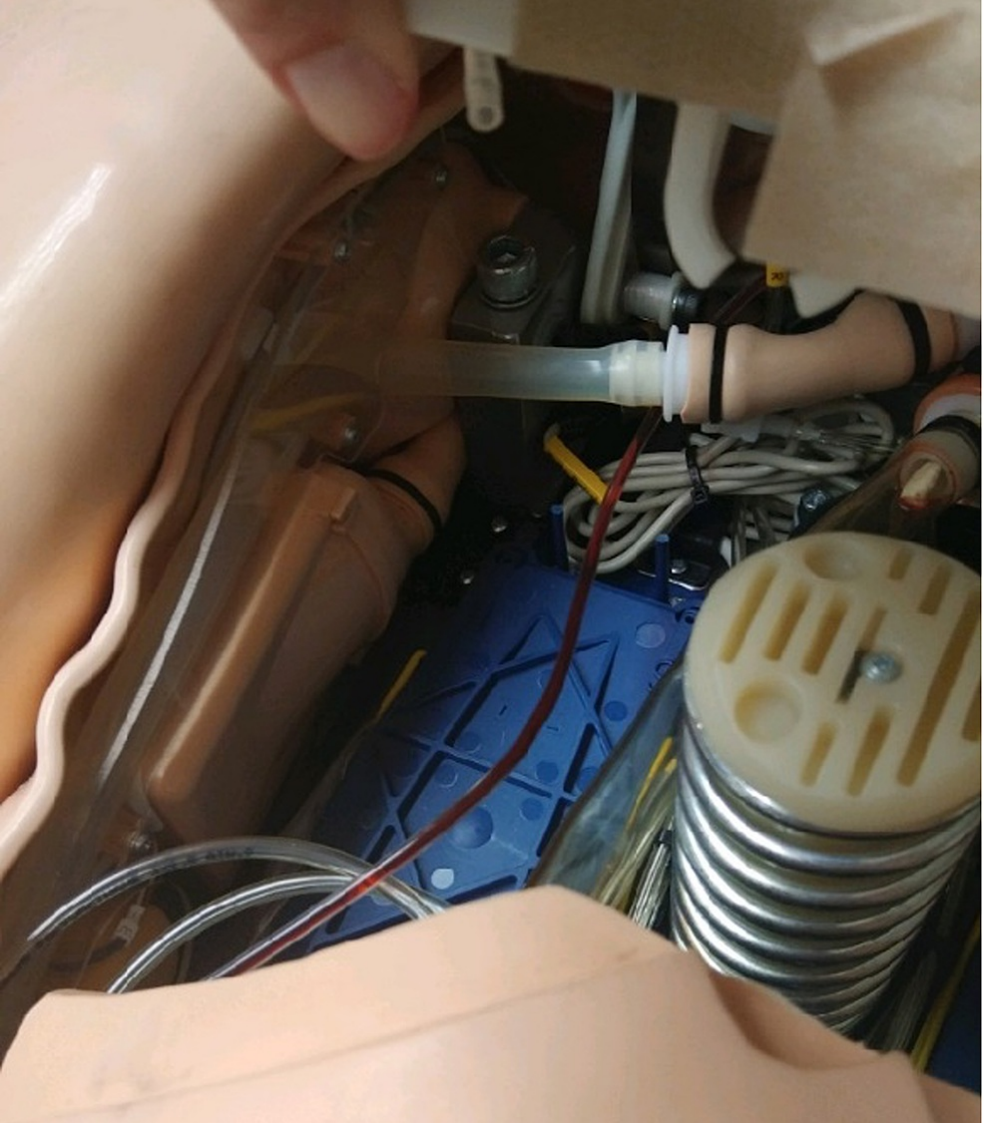
Intravenous tubing (red tubing line) running through the manikin interior

A pressure bag (Replaceable Pressure Infusor Bag™/Dual Setting Pressure Relief 1000 mL, Merit Medical, South Jordan, Utah, USA) and associated IV tubing should be obtained. The IV tubing should be run through the interior of the manikin’s body, terminating after just exiting into the simulated trachea through the previously drilled hole as demonstrated in Figure 2.

**Figure 3:**
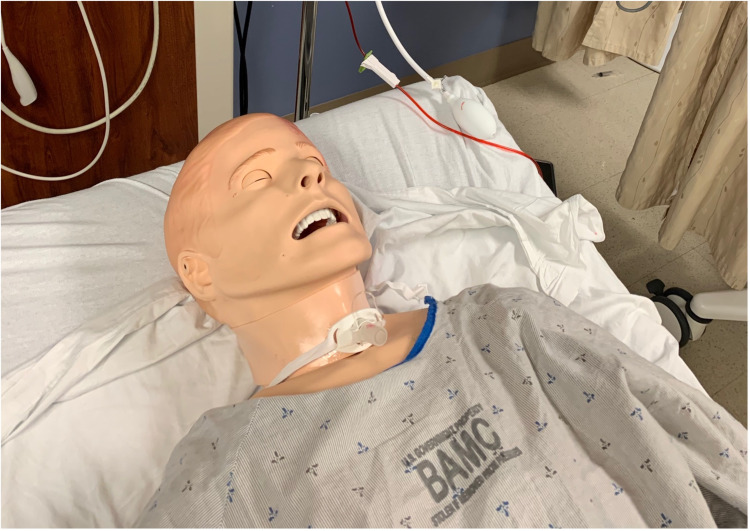
Tracheostomy tube in simulated tracheostomy site

At the level of the simulated trachea, a tracheostomy tube, Shiley™ Flexible Adult Tracheostomy Tube Cuffed with Disposable Inner Cannula (Medtronic PLC, Dublin, Ireland), was placed into the tracheostomy site on the mannequin as shown in Figure 3. Having an uncuffed tracheostomy tube in place at the beginning of the simulation is a good learning point, which would then require the learner to swap out the tube for a cuffed tube in order to provide cuff overinflation pressure. 

**Figure 4:**
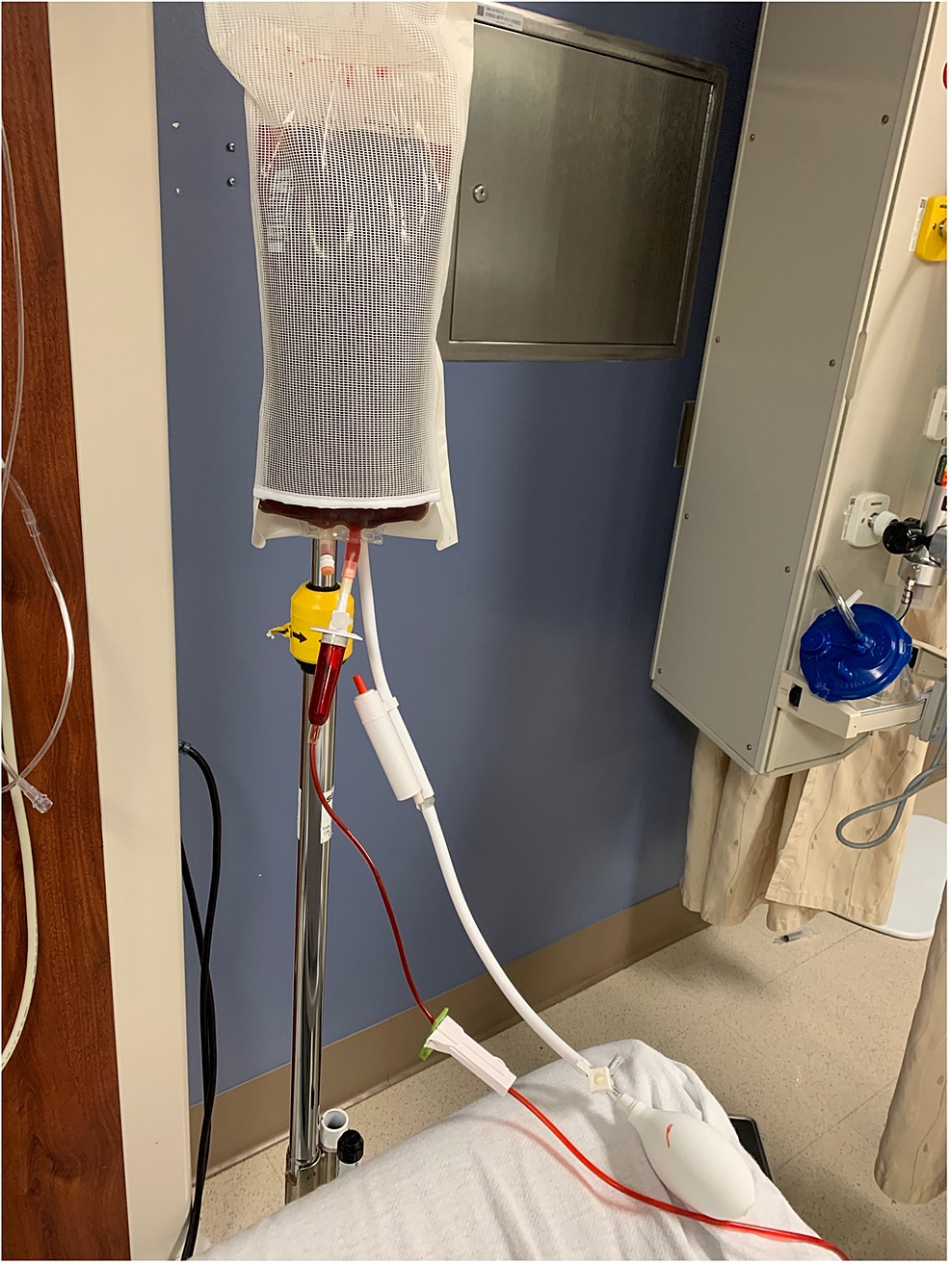
Demonstration of the simulated "blood" in the pressure bag system

A one-liter bag of IV fluids (Lactated Ringer’s Injection USP, Baxter Healthcare Corporation, Deerfield, Illinois) with red dye (McCormick® Red Food Color, 1fl oz, McCormick & Company, Baltimore, Maryland, or other food-grade red dye) added to create simulated blood should be attached to the IV tubing and placed in the pressure bag. Once ready for the simulation, the pressure bag can be manually inflated and deflated to increase or decrease the simulated hemorrhage as depicted in Figure 4. A more complex pneumatic device could be supplemented to pressurize the fluids but to keep this novel simulation low cost and simple to replicate we demonstrate the manual method. 

**Figure 5:**
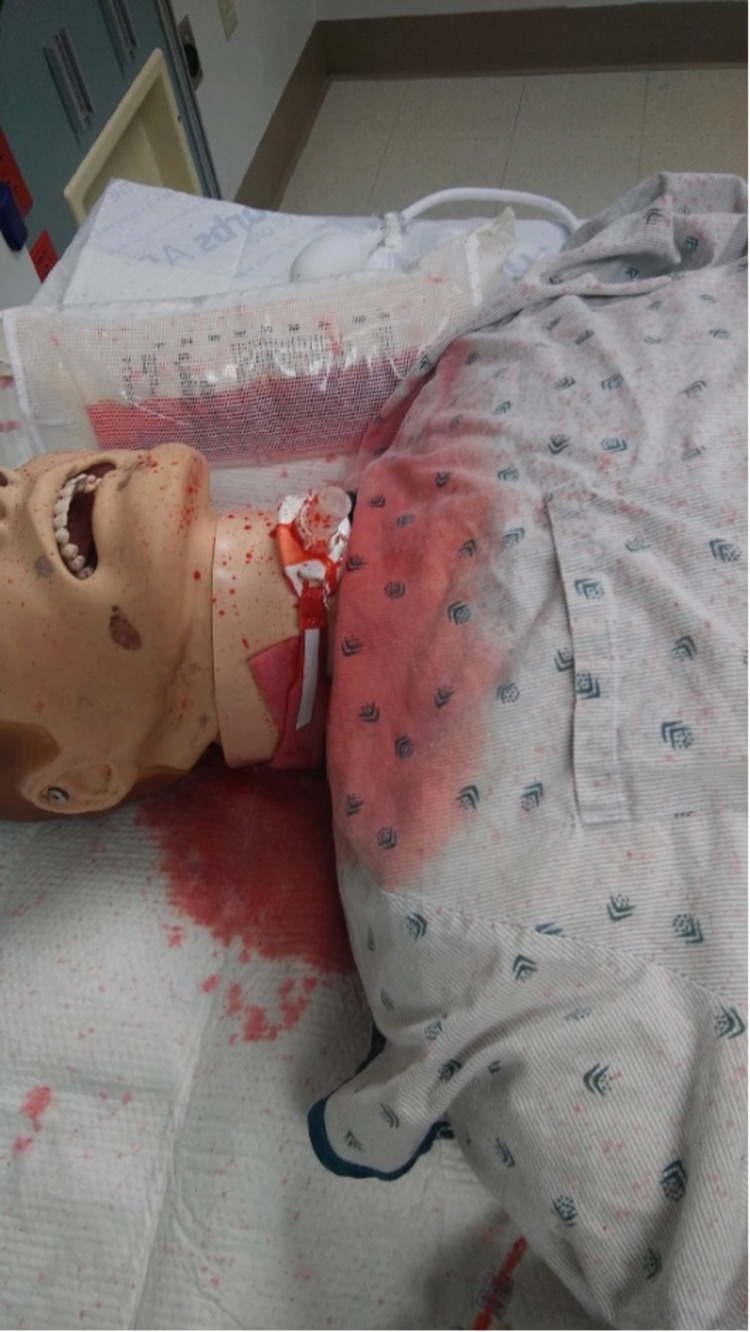
Final model with simulated tracheo-innominate artery fistula bleed

The final model is depicted in Figure 5, demonstrating how the simulation appears during use.

## Discussion

As an increasing number of patients are undergoing tracheostomies each year, complications associated with this procedure are more likely to be encountered. This is true for not only emergency medicine providers but for all providers involved in the care of these patients. Although TIFs have a high mortality rate, this can be substantially reduced if recognized and treated early [3,6]. 

This TIF bleed simulator can be utilized by simulation centers to incorporate this rare diagnosis into their simulation curriculum. This model has been integrated into the Brooke Army Medical Center (BAMC) Simulation Department’s training for the BAMC Emergency Medicine Residency. Residents have utilized this model in conjunction with a case-based simulation. The TIF model as described here is reusable and the simulator can be quickly and easily reverted back to its original state after simulation completion. 

## Conclusions

The low cost and ease of implementation of the modifications outlined in this article allow for the development of a novel TIF simulator that can be utilized to increase provider familiarity with a rare condition associated with high mortality. Providing clinical learners a simulated patient encounter improves a provider’s ability to rapidly recognize and appropriately treat patients presenting with this rare, life-threatening surgical emergency.
